# Using a Text Mining Approach to Explore the Recording Quality of a Nursing Record System

**DOI:** 10.1097/jnr.0000000000000295

**Published:** 2019-05-20

**Authors:** Hsiu-Mei CHANG, Ean-Weng HUANG, I-Ching HOU, Hsiu-Yun LIU, Fang-Shan LI, Shwu-Fen CHIOU

**Affiliations:** 1 MSN, RN, Department of Nursing, National Taiwan University Hospital, Taiwan, ROC; 2 PhD, Professor, Department of Information Management, National Taipei University of Nursing and Health Sciences, Taiwan, ROC; 3 PhD, RN, Assistant Professor, Department of Nursing, National Yang Ming University, Taiwan, ROC; 4 MSN, RN, Supervisor, Department of Nursing, National Taiwan University Hospital, Taiwan, ROC; 5 MSN, RN, Director, Department of Nursing, National Taiwan University Hospital Hsin-Chu Branch, Taiwan, ROC; 6 PhD, RN, Adjunct Associate Professor, School of Nursing, National Taipei University of Nursing and Health Sciences, Taiwan, ROC.

**Keywords:** nursing record, record quality, text mining

## Abstract

**Background:**

Most nursing records in Taiwan have been computerized, resulting in a large amount of unstructured text data. The quality of these records has rarely been discussed.

**Purpose:**

This study used a text mining method to analyze the quality of a nursing record system to establish an auditing model and associated tools for nursing records, with the ultimate objective of improving the quality of electronic nursing records.

**Methods:**

This study utilized a retrospective method to collect the electronic nursing records of 6,277 patients who had been discharged from the internal medicine departments of a medical center in northern Taiwan from January to June 2014. SAS Enterprise Guide Version 6.1 and SAS Text Miner Version 13.2 software were used to perform text mining. Nursing experts were invited to examine the electronic nursing records. The text mining results were compared against a benchmark that was developed by the experts, and the efficiency of SAS Text Miner was examined using the criteria of specificity, sensitivity, and accuracy.

**Results:**

In this study, 27,356 nurse-formulated events were used in the analysis. The results of the nurse-formulated events showed an 8.08% similar error with system-formulated events, 29.72% were identified as necessary and appropriate names, 17.53% were retained, 10.15% involved error event names, and 34.52% were not classified. In this study, the sensitivity of SAS text mining in the training (testing) data set was 96% (95%), and the specificity and accuracy were both 99% (99%).

**Conclusions:**

The results of this study show that text mining is an effective approach to auditing the quality of electronic nursing records. SAS Text Miner software was shown to identify inappropriate nursing record content quickly and efficiently. Furthermore, the results of this study may be included in in-service education teaching materials to promote the writing of better nursing records to improve the quality of electronic nursing records.

## Introduction

The computerization of medical information is a global trend. Increasingly, governments are facilitating the integration of information technology in healthcare and related settings. Using information systems allows patient information to be collected, retrieved, stored, and managed rapidly and accurately, and delivered instantly and correctly. It also effectively enhances team communication and enables patient-centered care. Nursing records are an important component of computerization and include information about patient physiological and psychological changes that nurses record in patient medical records after providing nursing care. These records are also critical legal documents representing the process of nursing care. Moreover, they are a crucial reference for clinical decision making (Almasalha et al., 2013; Feng, Tseng, Yan, Huang, & Chang, 2013; Paans, Sermeus, Nieweg, & Van Der Schans, 2010).

Nursing record computerization enhances the quality of medical records, reduces recording time, facilitates information sharing, and establishes standard nursing care guidelines, thus improving the efficacy of nursing work. Most medical institutions computerize nursing records using “nursing care plans” or “Focus Charting” record methods. In addition, a minority of institutions adopt an integrated approach that includes descriptive nursing records. Regardless of how the records are compiled, the content of nursing records should be objective, concise, clear, specific, immediate, organized, and accurate (Chang, Lee, & Lin, 2013; Hung, Han, & Lai, 2010; Wang, Yang, Sung, Liou, & Chen, 2011).

Information technology has been increasingly integrated into healthcare. Various research methods such as surveys of user attitudes toward the computerization of healthcare and nurse perceptions toward using computers in clinical settings have been used to evaluate nursing information systems. The results indicate that when recording templates or the interface design of the nursing information system does not meet user needs, user intention to use the system as well as the quality of information in the system may be affected (DeLone & McLean, 1992; Han, Kamber, & Pei, 2011; Yee et al., 2012).

In recent years, studies have applied data mining to select correct and useful information from large stores of data in electronic medical records. Information quality has been used as a criterion for nursing record management to improve the quality of medical care (Almasalha et al., 2013; Hunt, Sproat, & Kitzmiller, 2010; Lee, Lin, Mills, & Kuo, 2012). Text mining, a data mining approach, is used to search information to identify useful text fragments and further establish data analysis models, trends, or rules that may serve as the basis for monitoring information quality. For example, Kushima, Araki, Suzuki, Araki, and Nikama (2011) adopted a text mining approach to analyze the medical records of patients with chronic hepatitis. Relevant information was investigated by analyzing appropriate terms and feature words in nursing records to improve patient care quality (Byrne & Lang, 2013; Fayyad, Piatetsky-Shapiro, & Smyth, 1996; Park, Cho, & Chung, 2011).

Electronic nursing records may contain massive numbers of words. Record quality may be examined using a number of methods. For example, von Krogh, Nåden, and Aasland (2012) examined the record quality of a nursing information system based on the completeness, comprehensiveness, and coherence of the record content. Paans et al. (2010) applied the Delphi method to develop a D-Catch instrument, which they used to evaluate the quality of nursing records. Therefore, database quality management, which is the process of auditing and managing a large amount of electronic medical records, is a crucial topic in clinical nursing settings.

Recent studies on electronic nursing records have focused mostly on the evaluation of the developed system or on the degree of user satisfaction (Chang et al., 2013; Liao, Chu, & Chu, 2014; Wang et al., 2011). Few studies have investigated the quality of computerized nursing records. Therefore, this study used the text mining method to analyze the degree of accuracy with which the content of electronic nursing records complied with relevant hospital regulations, to determine the quality of these nursing records, and to propose a generally applicable model and set of tools to audit nursing records in order to facilitate improvements in recording quality.

## Methods

### Study Design

**Figure 1.**
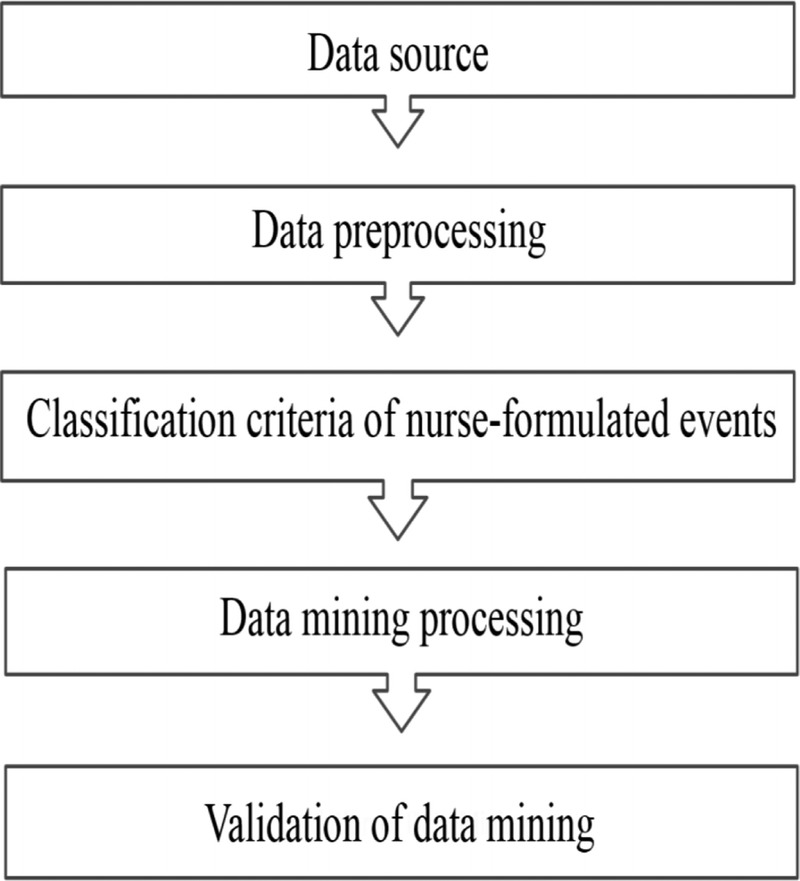
Flow chart of data processing and data mining.

A retrospective method was used to collect the electronic nursing records of 6,277 patients discharged from the internal medicine departments of a medical center in northern Taiwan from January to June 2014. The records of patients younger than 20 years old and patients positive for human immunodeficiency virus were excluded from the research samples. SAS Enterprise Guide Version 6.1 and SAS Text Miner Version 13.2 (SAS, Cary, NC, USA) software were used to perform text mining. The following sections describe data processing and data mining, and Figure 1 illustrates the related steps.

### Data Source

In this study, the basic patient demographic data set and diagnosis data set were obtained from the hospital information system, and data were obtained from the electronic nursing records of the nursing information system. Collected data included patient age, gender, diagnosis, and number of hospitalization days as well as subspecialty and nursing record. The electronic nursing record included the names of system-formulated events (SFEs), the nurse-formulated events (NFEs), and other event information with regard to nursing assessments, interventions, and outcomes.

The medical center targeted in this study identified health problems and events during the process of patient care and input this information into the electronic nursing record system to form electronic nursing records, which comprised the SFEs and NFEs that were chosen by the nurses. The process used to develop the nursing recording system was based on the principles or guidelines proposed in *On Call Principles and Protocols* edited by Marshall and Ruedy (2011). In this process, nursing staff of various wards were asked to provide lists of common health problems, which were then sorted systematically and used to build a system for the event. The nursing recording system was categorized into three record columns, including event name, nursing assessment and intervention, and evaluation/outcomes.

The SFEs comprised 93 types of events, which were categorized according to event classifications, including biological systems, nerves/muscles/bones, cardiovascular system, respiratory system, gastrointestinal system, skin, sensory perception, urinary system, psychological and social events, cancer treatment, obstetrics and pediatrics, value anomalies, patient tendencies, and common events. The nursing record system provided phrases regarding the nursing assessment, nursing intervention, and nursing outcomes related to a specific event, allowing nurses to select the appropriate phrases to write and edit in the record.

If no appropriate SFEs are available, nurses may add new events in blank fields based on patient conditions and edit the content of the events, which constitute the NFEs. As with the SFE, NFE content includes event name, nursing assessment and intervention, and evaluation/outcomes. The NFE comprises unstructured records and further includes a free-text column, in which nurses may edit any word.

### Data Preprocessing

Patient demographic and diagnosis information, as well as the electronic nursing record data file provided by the research hospital, were imported into a Microsoft Access database (Microsoft, Redmond, WA, USA). The data were transformed into a consistent format to build the data column required for research. Structured query language (SQL) was used to combine data files and process missing values. The data were then categorized, recoded, and input into SAS Enterprise Guide 6.1 and SAS Text Miner 13.2 for data mining and text mining.

The original data were collected from 235,798 electronic nursing records, of which 208,442 (88.40%) contained SFEs, indicating that a high proportion of nurses used the SFEs. In this study, 27,356 NFEs were used, accounting for 11.60% of the original data. This study analyzed the quality of the 27,356 NFEs using a text mining method.

### Classification Criteria for Nurse-Formulated Events

Three nursing experts were invited to examine the electronic nursing records and establish the classification criteria for the review of the NFEs in a meeting that discussed the hospital's nursing record writing regulations and guidelines and related literature. These nursing experts each had 10 years of working experience in cardiovascular, gastroenterology, endocrine, and hematology and oncology nursing, respectively and were responsible for nursing quality management and medical record reviews at the participating hospital.

**Figure 2.**
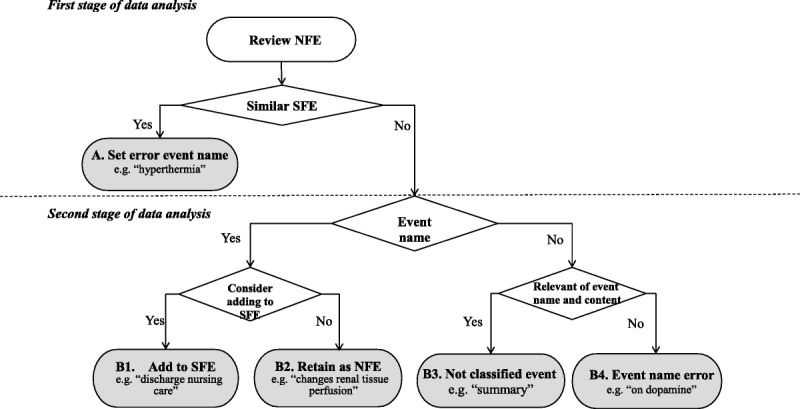
Nurse-formulated event (NFE) data analysis process. SFE = system-formulated event.

The review of nursing records for the NFEs was divided into two stages. Figure 2 shows the NFE data analysis process. In the first stage of the review, the experts determined whether an NFE was similar to any SFE. “Similar SFE” meant that different names were used to describe a very similar/same event in the SFE and NFE. In these cases, nurses should use SFE names rather than creating a new name. Therefore, this situation was classified as “Category A: Set error event name.” For example, when a nurse set a name in the NFE as “hyperthermia” and the event was similar to the “fever” event of SFE, this was regarded as an error of event similarity and was not reviewed again in the second stage.

When names of events differed from the SFE, the experts examined whether the event name was appropriate in the second stage. If the nurses described a new event name that had no equivalent SFE and was correct in terms of name and content, this event was classified as “Category B1: Add to SFE.” For example, the event names “discharge nursing care” and “examination and treatment” were considered appropriate to add to the system. Infrequent events that were not SFEs, but did not need to be included in the SFEs of the nursing record information systems, were classified as “Category B2: Retain as NFE.” An example of this would be when a nurse sets the name “changes renal tissue perfusion” to an event.

When nurses create an inappropriate NFE name, the experts analyzed the relevance of the event name to the content. If the NFE name was related to the content but the event could not be classified, it was categorized as “Category B3: Not classified event.” For example, nurses described a “summary” event that had an NFE name related to the content, but this summary was used as a substitute on the three shift nursing records. Finally, when NFE names or contents are irrelevant or provided with an incorrect event name, they are classified as “Category B4: Event name error.” For example, nurses created an “on dopamine” event name that was identified through expert review of the nursing record content and discussion; it was considered suitable to select the “shock and hypotension” of SFEs. In cases such as this, the nurse should use the SFE correctly and should not set an error event name.

Following these classification criteria helps determine the quality of nursing records. Categories B1 and B2 were considered “good quality” nursing records, and Categories A, B3, and B4 were considered “poor quality” nursing records. Nurses should try to use the SFE name to avoid using inappropriate names for events. These criteria were thus used as the data mining rules and algorithm.

### Data Mining Processing

This study performed text mining on 27,356 NFEs using SAS Text Miner 13.2 software. A random month was selected, and the data in that month were used as the training set. The data from all of the other months constituted the testing set. SAS Enterprise Guide 6.1 software was used to convert the data of 27,356 NFEs into SAS files, which were input in SAS Text Miner to establish a data source.

The text mining rules and algorithm were based on the expert review classification criteria (Figure 2), and text mining was conducted on the data of the training set and testing set using SAS Text Miner. The text mining results were compared with the benchmark, which is considered the gold standard of event classification. Our benchmark was developed by nursing experts, and the efficiency of SAS Text Miner was examined using criteria for specificity, sensitivity, and accuracy.

### Validation of Data Mining

The quality of the NFEs was evaluated based on expert review of nursing records. The three experts reviewed the training set separately and spent 8 weeks reviewing the nursing records of the training set as the gold standard for verifying training set data. However, because of limited time and manpower, only one nursing expert conducted the nursing record review for the testing set. The kappa statistic method was used to measure nursing expert interrater reliability, and these examination results were applied as the gold standard for verifying the results of SAS Text Miner.

The results of the two-stage review given by Experts 1 and 2 were identical, with each stage kappa value of 1.0. The results of Expert 3 and those of the other two experts were highly consistent, with the combined kappa values for the two stages of 0.98 and 0.99, respectively. The three experts discussed the items in 29 sets of data again to reach a consensus that served as the standard in the subsequent review of the testing set.

### Ethical Considerations

The requested data resource was retrieved from the database of the hospital's information center after personal information had been de-identified. This study was approved by the institutional review board of the target institution (no. 201408056RINC). The medical records were recoded through link removal. Only the data related to this study were selected for overall analysis, and no distinguishable personal information was present.

## Results

### Analysis of the Quality of Electronic Nursing Records

Nursing records should be objective, clear, timely, organized, and accurate. Electronic nursing records are clearer and more organized than traditional, handwritten records. In this study, the electronic nursing record quality was evaluated using a text mining method that includes the use of accurate event names and correlation between the event name and content.

The nursing records in the target hospital comprised SFEs and NFEs. In this study, 27,356 NFEs were used, accounting for 11.60% of the original data. The name and content of the NFEs were diverse and the terms used were varied. For example, the events for inserting an intravenous catheter were written as “ON CATH,” “ON CATHETER,” “ON IV CATH,” “inserting catheter,” “inserting intravenous catheter,” “catheter placement,” “placing catheter,” “placing intravenous catheter,” and “inserting tube.” Therefore, before data mining, it was necessary to clean, recode, and classify the NFEs to gain an accurate picture of the actual situation. As with the catheter example above, a large number of the NFEs were actually the same event but identified with disparate names.

**Figure 3.**
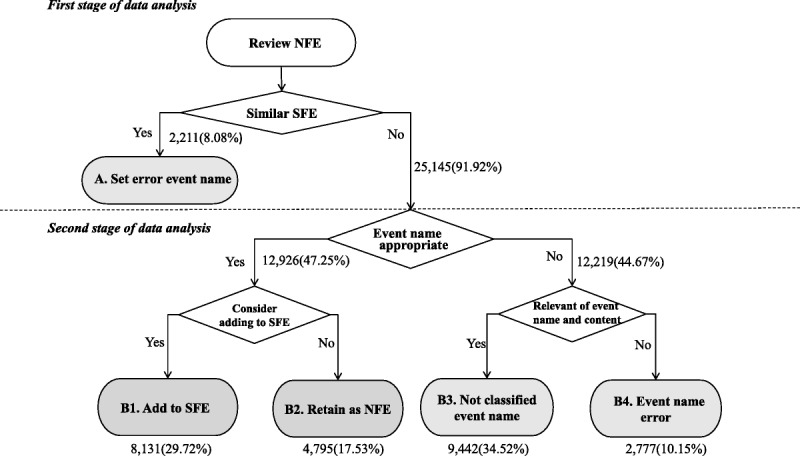
The results of the expert review of the nurse-formulated event (NFE). SFE = system-formulated event.

Figure 3 shows the results of the NFE distribution. The analysis of the quality of the NFEs found that the recording quality for Categories B1 and B2 were good, whereas the event names and contents of Categories A, B3, and B4 were poor and must be improved. The expert review revealed that 8.08% of the NFEs were errors due to their similarity with the SFEs (Category A), 29.72% could be added as new SFEs because the event names were associated with the content and the terms were correctly used (Category B1), and 17.53% could be retained as NFEs (Category B2) because in part of prior noninclusion in the system's list of SFEs.

Finally, 34.52% of the events were identified as having inappropriate names or as not classifiable (Category B3), and 10.15% had names that were irrelevant to the content or used incorrect names (Category B4). Overall, the results show that 47.25% of the NFE records were accurate (Categories B1 and B2); these NFE records were assessed as having good recording quality. Approximately 52.75% of the NFE records were assessed as having poor recording quality and contained Categories A, B3, and B4.

### Analysis of the Efficiency of SAS Text Miner

The experts established the rules for the text mining of the 3,800 NFEs in the training set. In the first stage, SAS Text Miner analyzed the level of similarity between the NFEs and the SFEs. The “event name” column was set as a variable, and the terms in the NFEs that were similar to the system-formulated terms were identified and set as the keywords for text mining. SAS Text Miner selected 463 NFEs that were similar to the SFEs. The analysis results found that the number of data analyzed correctly and incorrectly by the software was 441 and 27 events, respectively. Therefore, the sensitivity of SAS Text Miner was 96%, and the specificity and accuracy were both 99%.

The same text mining rules were applied to the 23,556 NFEs in the testing set. The SAS Text Miner selected 1,783 NFEs that were similar to the SFEs. The selected 1,783 events were compared with the 1,743 benchmark events identified by the experts, and the results showed that SAS Text Miner analyzed 1,649 events correctly and 134 events incorrectly. The sensitivity, specificity, and accuracy of SAS Text Miner for the training and testing sets are shown in Table 1, indicating that SAS Text Miner had high efficiency in analyzing the similarity between NFEs and SFEs. The testing set sensitivity of the software was 95%, and the specificity and accuracy were both 99%.

**TABLE 1.**
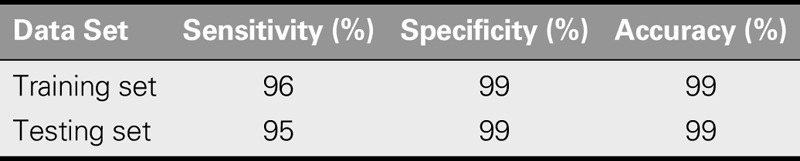
Sensitivity, Specificity, and Accuracy in the SAS Text Miner

If the NFEs differed from the SFEs, SAS Text Miner should determine in the second stage whether the event names were related to the content and accuracy of the terms used. The software is only able to search entire files using keywords, whereas using the “text filter snippet” mining function can filter related keywords and terms to select files related to the keywords. The content of these mining results must be examined by experts to determine the accuracy of terms and the level of association between the event names and the content. Therefore, the analysis conducted by SAS Text Miner in the second stage should be used only as a supplementary tool for audits of nursing record quality.

## Discussion

### Quality of Electronic Nursing Record

Of the electronic nursing records examined in this study, 88.40% used SFEs. Using a customized nursing record information system enhanced the association between nursing record content and the completeness and clarity of the records. Overall, the quality of nursing records improved. Approximately 11.60% of the NFEs were created by nurses because they did not find appropriate SFEs for use in the nursing record.

According to the results, 29.72% of the NFEs may be added directly into groups of SFEs with the correct use of event names. It is recommended that the hospital use common NFEs as SFEs with consistent terms to prevent nurses from using inconsistent terms or inappropriate abbreviations for the same NFE. Examples of this include “wound care,” “discharge nursing care,” “invasive examination and treatment,” and “drug dosage adjustment.”

This study analyzed the quality of NFEs and found that 17.53% of the events were appropriate for retention in their current nurse-formulated form—a result that is consistent with Lee (2006) and Yee et al. (2012). When an event could not be recorded using standardized nursing language, nurses adapted accordingly, indicating that a nursing record system that provides NFEs promotes the flexibility and convenience needed for nurses to compose nursing records.

However, if the NFE is similar to the SFE, the data in the nursing recording system database may be inconsistent, which will affect future data analysis. In this study, 8.08% of the NFEs were classified as the error event name being similar to SFEs. For example, NFEs that included terms such as “respiratory wheezing,” “wheezing,” “wheezing during activity,” and “gasping for breath” were actually describing the same event as the SFE “shortness of breath.” In addition, 10.15% of the NFEs were categorized into error event name categories. For example, nurses should not create NFE of “on dopamine,” because it belongs to event category of the cardiovascular system “hypotension and shock” in the SFE.

This study performed only data mining and did not investigate the real experience of nurses using the nursing record system. Therefore, the reasons proposed for our results are only suggested as being the same as those for not using the recording templates of nursing record system in Wang et al. (2011); that is, the nurses felt that making their own records was faster and more convenient than using record templates provided in the system.

The nursing record system used in the target hospital was unable to identify errors of similarity between NFEs and SFEs. According to the results, as shown in Figure 3, poor-quality nursing records, including Categories A, B3, and B4, accounted for approximately 52.75% of the NFEs. Thus, hospitals should provide these inadequate record examples for in-service nursing education and train nurses to understand how to use SFEs correctly to reduce errors and promote clarity in nursing records.

In addition, the nursing department should establish a standard for regular audits of electronic nursing records to ensure compliance and improve the quality of electronic nursing records. Moreover, when performing system updates, the hospital should either add a function to search for synonyms of system-formulated terms or provide experts with the function of automatic assessment to reduce errors of event similarity and data inconsistency.

In this study, nursing staff of different divisions used a variety of terms to record the same nursing care activity. For example, “abdominal tapping” was also termed “abd tapping,” “paracentesis,” and “abdominal paracentesis drainage.” This result is consistent with that of Wu, Chen, and Chen (2009) and Yee et al. (2012), who indicated the difficulty with use of standardizing nursing language. This study analyzed NFEs and found that content used Chinese, English, uppercase and lowercase English letters, and various medical terminologies intermittently.

The original data were examined manually to determine whether the same nursing activities and terms could be collated, reclassified, and coded for subsequent data interpretation. In other words, data processing entailed a significant amount of time and energy. Therefore, as suggested by Feng et al. (2013), structured and coherent languages and methods comprise a critical base and foundation for establishing medical information systems.

### Text Mining Software-Facilitated Auditing of the Quality of Nursing Records

Paans et al. (2010) adopted the D-Catch instrument as a tool to assess nursing records. However, their process of assessment was complex and time consuming. Text mining provides information search, association, prediction, analysis, management, and decision-making support for a rapid and accurate analysis of unstructured text (Han et al., 2011; Liao et al., 2014; SAS Resource Center, 2014). This study employed SAS Text Miner to conduct text mining on electronic nursing records, identified the information hidden in the NFEs, and determined a method for auditing the quality of electronic nursing records. The results were consistent with those of Kushima et al. (2011), which suggested that text mining may be used as a basis for improving medical care quality.

In addition, this study used SAS Text Miner to analyze the quality of electronic nursing records and found the software to have high sensitivity, specificity, and accuracy in both the training and testing data sets. SAS Text Miner efficiently and accurately analyzed the NFEs that were similar to SFEs. Moreover, its “text filter snippet” mining function permitted the selection of words with high association coefficients and identified related documents.

In this study, 34.52% of the NFEs were identified as having inappropriate event names. For example, “summary” was used to refer to the nursing records of three shifts. Nursing expert analysis of the events described by “summary” found a diverse range of contents. SAS Text Miner performed text mining on the 9,442 events of “summary” and identified the events related only to SFEs within a few minutes.

The results of this study indicated that SAS Text Miner has a fast and effective text mining tool and supported text mining as a strategy to reduce the amount of time spent on manually examining electronic nursing records. Therefore, this software package can be used as an auxiliary tool for auditing electronic nursing records.

### Conclusions

This study analyzed the association between the NFEs and the SFEs and found that 8.08% of the NFEs had an error of being similar to SFEs. Among the NFEs, 29.72% of the terms could be added as new SFEs because their content was correct, and 17.53% should be retained as NFEs. This result may be referenced by professionals who design and implement updates of nursing record systems. In addition, 10.15% of the NFEs were classified as the error category of “incorrect event name,” and 34.52% of the event names could not be adequately classified. This result may serve as a teaching material reference for in-service education related to nursing records in nursing record systems.

This study conducted text mining on NFEs using SAS Text Miner. The sensitivity, specificity, and accuracy of SAS Text Miner for the training set were 96%, 99%, and 99%, respectively, whereas those for the testing set were 95%, 99%, and 99%, respectively. These results indicate that SAS Text Miner is an excellent audit tool for electronic nursing records.

### Implications for Nursing Practice

The recommended applications of the findings of this study in future clinical nursing include the following:

1. By evaluating the nursing recording system and the text mining model of NFEs, it is possible to identify NFEs that are commonly used by nurses as well as those that should be added as SFEs. In addition, the nursing recording system should enhance the search function and set the list of common SFE names for various units to improve nursing record consistency and accuracy.
2. In-service education on electronic nursing records should be provided for nursing staff. Nursing documentation teaching materials should discuss the SFE terms that are typically incorrectly chosen (resulting in NFE names that are similar to SFEs), NFEs that are difficult or currently impossible to classify, and event names that are selected incorrectly. In-service education should promote the correct use of structured SFE names to enhance the consistency and accuracy of nursing records and the regular audit of nursing records to improve the quality of nursing records.
3. SAS Text Miner software is able to quickly identify relevant items from a large number of documents, reducing the time and cost involved in manual reviews of medical records. It is suggested that SAS Text Miner be used as a tool for nursing record quality management.
4. In the future, the text mining model proposed in this study may be applied to the unstructured text forms of data files of medical institutions and used as a way to audit the quality of electronic medical records.

### Limitations

This study targeted only the nursing records of patients in the internal medicine wards of one medical center in northern Taiwan because of limitations in time, manpower, and material resources. Moreover, as electronic nursing record system designs vary from hospital to hospital, further verification is necessary to determine whether this research method can be used in other hospitals. In this study, a retrospective electronic data analysis was conducted to evaluate the quality of nursing records in terms of accuracy, association, and completeness of content, but the timeliness of the records could not be evaluated. Although SAS Text Miner was used in this study, other data mining software such as IBM DB2 Intelligent Miner and PolyAnalyst are available. Therefore, future studies may also investigate the efficiency of other text mining software.
